# Multi-Criteria Decision Analysis for Assessment and Appraisal of Orphan Drugs

**DOI:** 10.3389/fpubh.2016.00214

**Published:** 2016-09-30

**Authors:** Georgi Iskrov, Tsonka Miteva-Katrandzhieva, Rumen Stefanov

**Affiliations:** ^1^Department of Social Medicine and Public Health, Faculty of Public Health, Medical University of Plovdiv, Plovdiv, Bulgaria; ^2^Institute for Rare Diseases, Plovdiv, Bulgaria

**Keywords:** health technology assessment, reimbursement, decision-making, multi-criteria decision analysis, orphan drugs, rare diseases

## Abstract

**Background:**

Limited resources and expanding expectations push all countries and types of health systems to adopt new approaches in priority setting and resources allocation. Despite best efforts, it is difficult to reconcile all competing interests, and trade-offs are inevitable. This is why multi-criteria decision analysis (MCDA) has played a major role in recent uptake of value-based reimbursement. MCDA framework enables exploration of stakeholders’ preferences, as well as explicit organization of broad range of criteria on which real-world decisions are made. Assessment and appraisal of orphan drugs tend to be one of the most complicated health technology assessment (HTA) tasks. Access to market approved orphan therapies remains an issue. Early constructive dialog among rare disease stakeholders and elaboration of orphan drug-tailored decision support tools could set the scene for ongoing accumulation of evidence, as well as for proper reimbursement decision-making.

**Objective:**

The objective of this study was to create an MCDA value measurement model to assess and appraise orphan drugs. This was achieved by exploring the preferences on decision criteria’s weights and performance scores through a stakeholder-representative survey and a focus group discussion that were both organized in Bulgaria.

**Results/Conclusion:**

Decision criteria that describe the health technology’s characteristics were unanimously agreed as the most important group of reimbursement considerations. This outcome, combined with the high individual weight of disease severity and disease burden criteria, underlined some of the fundamental principles of health care – equity and fairness. Our study proved that strength of evidence may be a key criterion in orphan drug assessment and appraisal. Evidence is used not only to shape reimbursement decision-making but also to lend legitimacy to policies pursued. The need for real-world data on orphan drugs was largely stressed. Improved knowledge on MCDA feasibility and integration to HTA is of paramount importance, as progress in medicine and innovative health technologies should correspond to patient, health-care system, and societal values.

## Introduction

### HTA and Reimbursement Decision-Making

Ongoing progress in medicine, sustaining financial burden on health-care systems, and increasing demand for public scrutiny have made authorities and payers rewrite health policy over the last decade. With growing use and importance of health technology assessment (HTA) in reimbursement decision-making, stakeholders are confronted with methodological challenges due to specific characteristics of health technologies, their developmental environment, and regulation process (1).

Health technology assessment is defined as a multidisciplinary process that summarizes information about the medical, social, economic, and ethical issues related to the use of a health technology in a systematic, transparent, unbiased, robust manner. In times of fiscal austerity, HTA has been a particularly appraised decision support tool for formulation of safe, effective, health policies that are patient focused and seek to achieve best value (2). HTA is usually perceived to be weighting clinical and economic evidence, combining those considerations into an incremental cost-effectiveness ratio (ICER). Theoretically, this outcome should define reimbursement decisions. Nevertheless, ICER and HTA remain purely technical tools. It is the health authorities that make final decisions, taking into account various other factors as well. Innovative health technologies complicate this problem further. They require additional consideration of context and implementation attributes, which go far beyond ICER (3, 4).

Limited resources and expanding expectations push all countries and all types of health systems to adopt new approaches in priority setting and resources allocation. All stakeholders understand that decision-making is a complex process. Despite best efforts, it is difficult to reconcile all competing interests, and trade-offs are inevitable. This explains the recent uptake of value-based pricing and reimbursement (5). Furthermore, innovation in medical research and development requires innovation of the HTA process too. HTA should be updated in order to respond to such challenges, as innovative health technologies pose new critical factors, which affect patients, payers, and providers (6). Current HTA methodology presents limitations regarding integration of the diversity of stakeholders’ perspectives in reimbursement decisions. Standard health economic tools do not allow responding to those concerns. A possible way to address this issue would be to have a societal consensus on a collective solution for all stakeholders (7). This could be achieved by engaging all groups and reflecting all preferences.

### MCDA and Reimbursement Decision-Making

Multi-criteria decision analysis (MCDA) could be a major factor in this health policy challenge. This methodology represents a process of evaluation of alternatives by taking into account multiple criteria in an explicit manner. MCDA provides a structured, transparent approach to identify preferred alternatives by means of combined calculation of the relative importance of different criteria and the performance of the alternatives on these criteria. MCDA framework enables the exploration of stakeholders’ preferences, as well as the explicit organization of broad range of criteria on which real-world decisions are based. MCDA gives insights into the rationale behind value assessment and appraisal (5, 8).

Health technology assessment and reimbursement decisions are complex due to multiple aspects considered and gaps in evidence. However, transparency is not about uniformity of decisions. Transparency means consistency of decisions over time. Decision-making framework needs to explain how seemingly different appraisals are done by different groups at different times. HTA inevitably goes together with limits. Nevertheless, restrictions are most accepted when they are transparent and consistent. Reimbursement decisions should foster sustainable population health by recognizing policy priorities and fiscal constraints while giving due weight to the rights and claims of individuals who seek health care (9).

### Reimbursement Decision-Making of Orphan Drugs

Innovative therapies present an excellent opportunity for a case study of the integration between HTA and MCDA. Assessment and appraisal of these medicinal products tend to be one of the most complicated HTA tasks. This assumption is particularly viable in regard to rare diseases and orphan drugs (10–13). Rare diseases represent a unique challenge to health authorities and payers, as they are life-threatening or chronically debilitating conditions with a low prevalence and a high level of complexity. It is estimated that between 5000 and 8000 distinct rare diseases affect 27–36 million people in the European Union (EU). Because of this magnitude, rare diseases call for special, combined efforts to prevent significant morbidity or avoidable premature mortality and to improve quality of life and socioeconomic potential of affected persons (14).

Despite regulatory incentives that have stimulated research and development of orphan drugs at a global level, timely access to market approved ones remains an issue. There are legitimate concerns among stakeholders that access to innovative therapies for rare diseases is greatly delayed. Conventional HTA is mainly concentrated on ICER, marginalizing all other considerations. Value assessment and appraisal of orphan drugs is, however, a debate of policy priorities, health system specifics, and societal attitudes. HTA bodies and payers need to pursue a multidisciplinary analysis on a range of criteria, ensuring an explicit understanding of trade-offs in reimbursement decisions. It is also important to address the impact of rarity, as quality of orphan drug evidence is not the same as for conventional therapies. Early constructive dialog among rare disease stakeholders and elaboration of orphan drug-tailored decision support tools could set the scene for ongoing accumulation of evidence, as well as for proper and timely value assessment and appraisal (15, 16).

### Aim of the Study

The objective of this study was to create an MCDA value measurement model to assess and appraise orphan drugs. This research built upon two previous studies in Bulgaria – one analyzing reimbursement decision-making on orphan drugs under current HTA legal framework (17) and second identifying important criteria that are relevant to local stakeholders (4). Therefore, the present study’s aim was achieved by exploring the preferences on decision criteria’s weights and performance scores through a stakeholder-representative survey and a focus group discussion.

## Materials and Methods

### Design of the Value Assessment and Appraisal Model

Multi-criteria decision analysis process consists of several steps: definition and structure of the problem; identification of a set of decision criteria; elicitation of decision criteria’s weights and performance scores; and final value estimate (7). A number of MCDA methodologies are available, with various degrees of complexity. We used a simple MCDA linear additive model. This methodology was chosen as being of most value to health authorities and payers (11). The model calculates an overall value combining weighted performance scores for all relevant criteria. Value assessment and appraisal took a societal perspective. It was initially decided that our MCDA model would be 100-point, with 100 points being the best possible outcome.

### Identification of a Set of Decision Criteria

The initial list of reimbursement decision criteria was made through a previous closed-ended survey among four stakeholder groups (medical professionals, patient representatives, health authorities, and industry representatives), reported elsewhere (4). This study yielded 15 criteria to form a tentative optimal model for drug reimbursement decision-making. Following a cross-comparison with the current legal framework in Bulgaria (17), those preliminary criteria were reorganized into three categories: health technology’s characteristics (five criteria), indicated disorder’s characteristics (two criteria), and public health aspects (five criteria). All but two criteria (disease burden and budget impact) were qualitative. In order to define different performance scores for each of those qualitative criteria, specific cases and scenarios were identified from a literature review, with a specific focus on existing payer value assessment and appraisal frameworks.

### Elicitation of Decision Criteria’s Weights and Performance Scores

Elicitation of weights and performance scores was done through an online survey in January–March, 2015. Questionnaire consisted of 19 questions, grouped into sociodemographic profile, weight elicitation, performance score estimation, and additional contextual considerations. Participants were provided with definitions of all criteria and scales used in the survey. In order to be validated, the questionnaire was pretested by six participants.

Weight elicitation aimed to catch stakeholder preferences of what is most important in reimbursement decision-making and which criteria should contribute most to value assessment and appraisal. A two-step 100-point weight elicitation technique was used. First, respondents were asked to distribute 100 value points among the three decision criterion categories. Higher number of points meant higher importance and more weight in the overall value estimate. Participants had to provide relative weights for each criterion category from their individual perspective, but in the context of reimbursement decisions in general. Then, participants had to distribute 100 value points among the criteria of each category. Individual weights of criteria were normalized to sum up to the weight of the criterion category that was elicited at step one.

Performance score elicitation aimed to understand variations on how a health technology is assessed and appraised with regard to its outcome for each decision criterion. A similar technique was applied. Participants had to evaluate different predefined performances for each criterion. Rating scale for performance scores ranged from 0 points (worst, least desired outcome) to 100 points (best, most desired outcome). Final weighted score of each performance was calculated as the percent from the normalized weight (in points) of the corresponding criterion.

### Pilot Value Assessment and Appraisal Model

The preliminary model based on survey results was piloted during a focus group discussion in September, 2015. Authors of this paper acted as facilitators. Participants were given the survey results in advance. The discussion was structured. At the beginning of the meeting, attendees were reminded about the study’s aim and nature. They were offered the opportunity to revise the list of criteria, weights, and performance scores. Any changes if necessary, however, had to be justified and consensually agreed by all.

Then, in order to test the overall model, participants had to rate two case studies of hypothetical orphan drugs. The two medicinal therapies were indicated in two hypothetical rare diseases with clearly differentiated profiles (Table 1). This approach was undertaken, as preliminary survey results showed disease severity to be the single most important decision criteria.

**Table 1:** **Case studies for the pilot model testing**.

Rare disorder’s characteristics	Orphan drug A	Orphan drug B
Prevalence	<1 in 10,000 (ultra rare disorder)	1–5 in 10,000 (rare disorder)
Onset	Onset in childhood	Mixed onset
Need for carer	Strong need for carer (severe physical and/or mental impairment)	Mild need for carer (mild physical impairment, no mental impairment)

Within the focus group discussion, stakeholders had to assess and appraise both orphan drugs, applying the value measurement model. Ratings for each criterion were discussed and consensus performance scores served as a “realistic” assessment and appraisal scenario. Cases, when agreement was not reached, provided scores for “pessimistic” (lowest total value estimate) and “optimistic” (highest total value estimate) scenarios. Aided by these six scenarios for the two orphan drugs, participants were asked to decide upon a reimbursement recommendation threshold.

### Study Participants

Study participants included four groups of public health stakeholders from Bulgaria: medical professionals, heading university hospital clinics, chairs of rare disease patient organizations, health authorities (reimbursement decision-makers, working at the macro level), market access and governmental affairs executives of pharmaceutical companies. A total of 307 stakeholder representatives were contacted by e-mail to participate in the survey with an invitation letter describing the study. Participants were identified through past or present participation (prior works, publications, positions held, etc.) in decision-making on drug reimbursement with public funds in Bulgaria. Respondents were asked to take their own perspective into account when providing the relative importance of decision criteria and appraising different performances for achievement of those indicators.

Four representatives of each stakeholder group were invited to attend a focus group discussion. These individuals were deliberately selected from the survey respondents. They were additionally chosen to assure variation with regard to age, sex, geography, pathology, and governance type.

Approval by Ethics committee was not required for this research. The survey and the focus group discussion were sociological from a methodological point of view, with no clinical research. No personal data were saved or analyzed.

### Data Analyses

Weights and performance scores obtained from the survey were analyzed using SPSS (version 11.5; SPSS, Inc., Chicago, IL, USA). Descriptive statistics were applied. Differences in mean weights and performance scores were compared between stakeholder, age, and sex groups. As data were not normally distributed, chi-square and Kruskal–Wallis tests were applied to determine whether variations between the groups were significantly different at 0.05 levels.

## Results

### Participants

In total, 143 participants completed the survey, 46.6% overall response rate (Table 2). Medical professionals and industry representatives produced the biggest numbers of respondents, while response rate was higher in patient and health authority groups. Also, 11 participants (3.6%) declined to complete the survey.

**Table 2:** **Response rate per stakeholder groups**.

Stakeholder groups	Survey completed (%)	Decline to participate (%)	Without response (%)	Total
Medical professionals	41 (36.3%)	–	72 (63.7%)	113
Patient representatives	31 (57.4%)	–	23 (42.6%)	54
Health authorities	32 (56.1%)	4 (7.0%)	21 (36.9%)	57
Industry representatives	39 (47.0%)	7 (8.4%)	37 (44.6%)	83
Total	143 (46.6%)	11 (3.6%)	153 (49.8%)	307

Respondents aged 36–45 years constituted the biggest group of participants (57 people). Also, 63% of the surveyed were women who dominated substantially among medical professionals, health authorities, and patient representatives. Mean duration of professional experience in the field of public health and/or rare diseases was 12.7 ± 9.2 years. Older respondents, medical professionals, and patient representatives tended to have a longer experience.

### Relative Weights of Decision Criterion Categories

**Figure 1:**
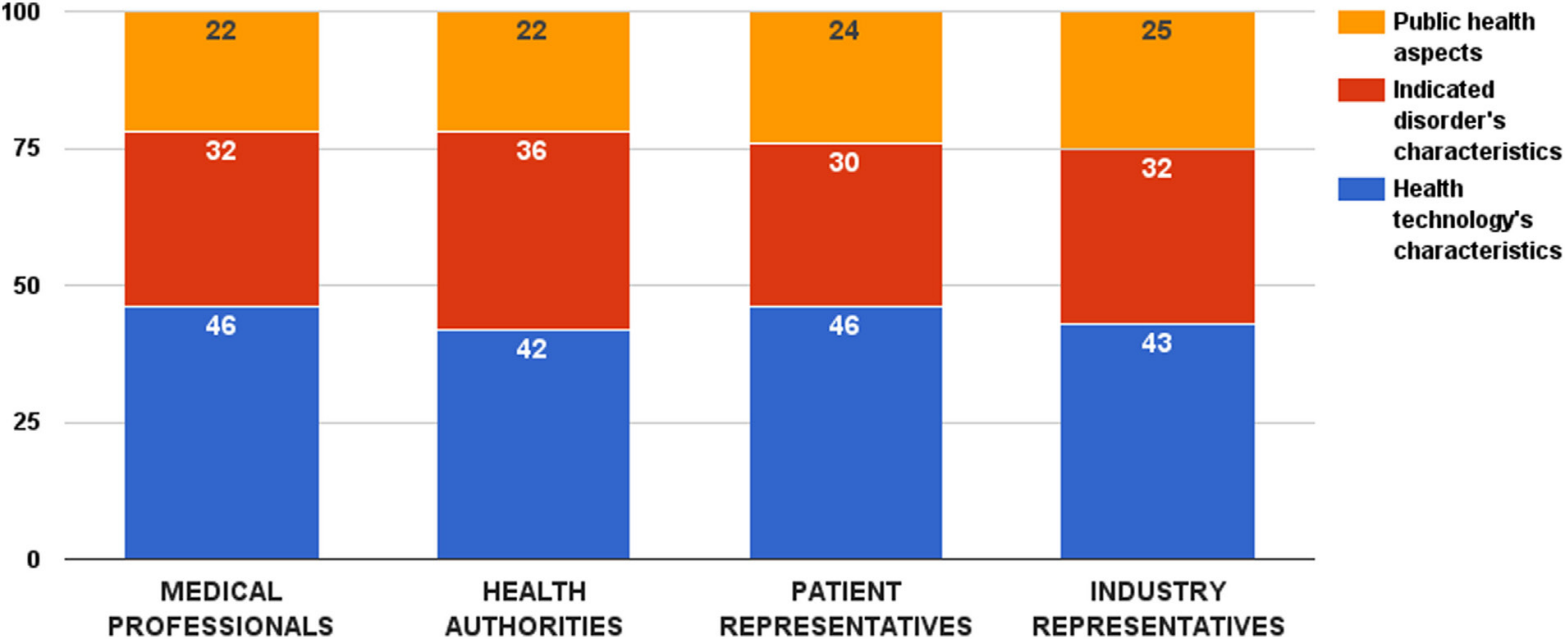
**Relative weight of decision criterion categories per stakeholder groups**.

Survey participants provided highest relative weight to the decision criterion category that accounts for the properties of the health technology – 44 ± 13 value points out of 100. No significant difference was observed among stakeholder groups (*Z*_Kruskal–Wallis_ = 4.09, *p* = 0.252) (Figure 1). Other two decision criterion categories received 32 ± 11 (criteria describing the indicated disorder) and 24 ± 10 points (criteria accounting for the public health considerations), respectively. Again, there was no significant difference in these weights among the surveyed groups (*Z*_Kruskal–Wallis_ = 5.12, *p* = 0.163 and *Z*_Kruskal–Wallis_ = 1.55, *p* = 0.671, respectively).

### Weights and Performance Scores of Decision Criteria That Describe the Health Technology’s Characteristics

**Figure 2:**
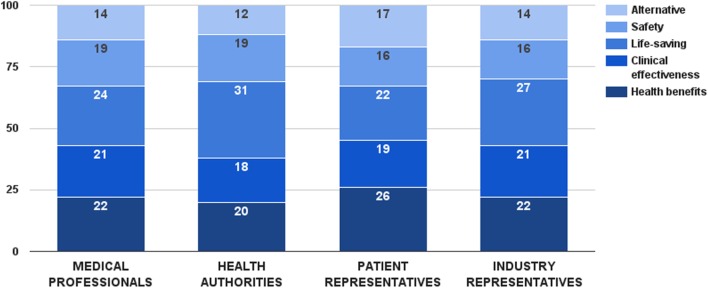
**Relative weight of decision criteria that describe the health technology’s characteristics per stakeholder groups**.

Survey participants considered life-saving as the most important decision criterion from this category. This criterion received 26 ± 12 value points out of 100, followed by health benefits and clinical effectiveness. There was a general consensus between stakeholders regarding the values of these criteria, except for life-saving (Figure 2). In this particular case, health authorities gave a significantly higher weight to this consideration (*Z*_Kruskal–Wallis_ = 8.11, *p* = 0.044). Individual weights of all five criteria were later normalized to sum up to the weight of the criterion category (44 points). Final weights of these criteria were: health benefits – 10 points, clinical effectiveness – 9 points, life-saving – 11 points, safety – 8 points, and alternative – 6 points.

Different performance scores for the health benefits criterion were defined based on health technology’s impact on life expectancy and quality of life. Cure was the ultimate performance, receiving the maximum score of this criterion – 10 points. Combination of extending life expectancy and improving quality of life was considered next best option, estimated with the same amount of points by the stakeholders. Choosing between life expectancy and quality of life, improving quality of life received 7 points, one point more than extending life expectancy. Scores for the clinical effectiveness criterion were differentiated through achievement of clinical and statistical significance. Combination of both options was the best case, getting the maximum 9 points for this criterion. Performance of clinical significance was preferred over performance of statistical significance – 5 vs. 2 points, respectively. Life-saving and alternative criteria were constructed as dichotomous variables, getting all the points in the maximum case and no points at all in the minimum case.

### Weights and Performance Scores of Decision Criteria That Describe the Indicated Disorder’s Characteristics

**Figure 3:**
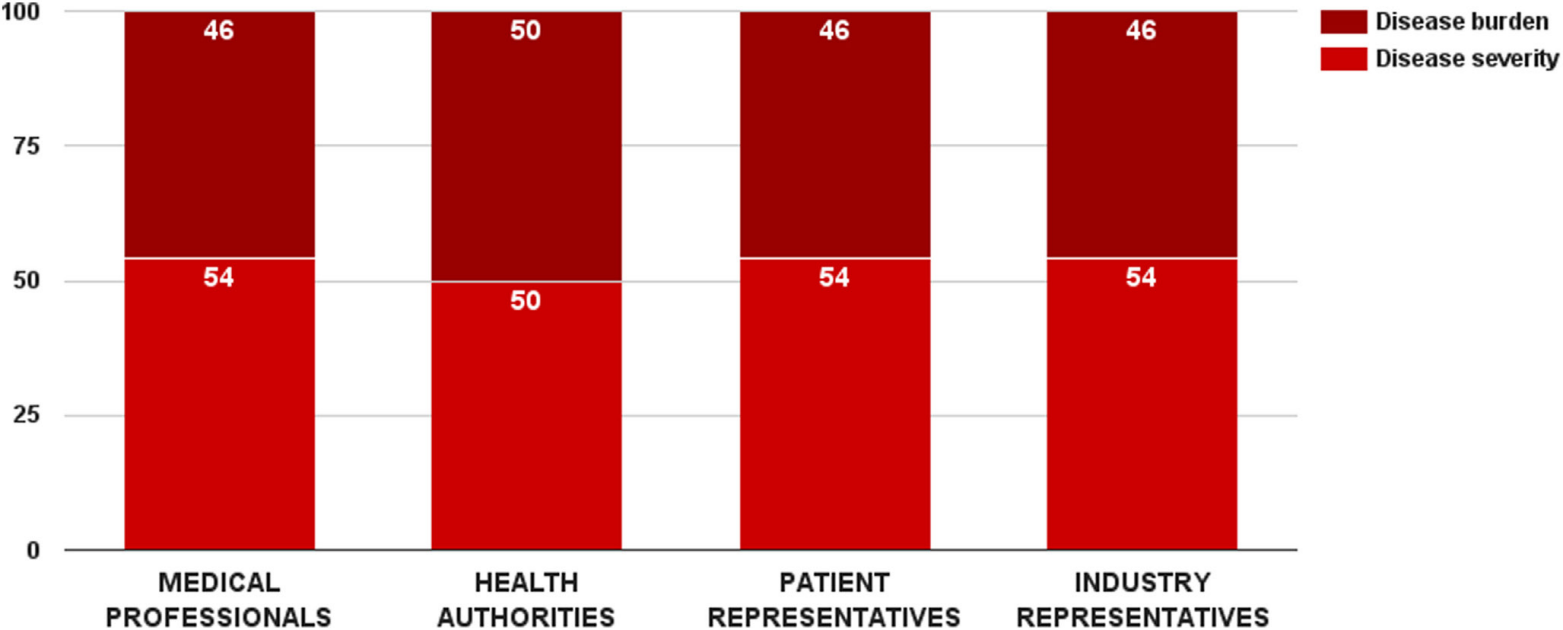
**Relative weight of decision criteria that describe the indicated disorder’s characteristics per stakeholder groups**.

Survey participants gave a slight preference to disease severity over disease burden. The disease severity criterion received 53 ± 14 value points out of 100 for this category. There was a general consensus between stakeholders regarding the weights of these two criteria (Figure 3). Medical professionals, patient, and industry representative considered disease severity more important in reimbursement decision-making, while health authorities was the only group that rated the two criteria equally. Nevertheless, there was no significant overall difference in the weight values per surveyed groups. Individual weights of the two criteria were normalized to sum up to the weight of the criterion category (32 points). Final weights of these criteria were disease severity – 17 points and disease burden – 15 points.

Scores for the disease severity criterion were differentiated through disease progression and prognosis. Estimated performance scores for these scenarios were affected by the study’s focus on rare diseases. Stakeholders gave more importance on chronic life-threatening conditions than on acute ones – 17 vs. 15 points. These results were not unanimous. Overall, medical professionals and industry representatives considered chronic life-threatening disorders more severe, while health authorities preferred acute conditions. The difference in the performance scores per groups was significant for both cases (*Z*_Kruskal–Wallis_ = 15.24, *p* = 0.002 for acute disorder and *Z*_Kruskal–Wallis_ = 16.32, *p* = 0.001 for chronic life-threatening disorder).

Disease burden was one of the two criteria in the study whose performance could be quantified. Raw data were taken from the results of an EU-funded project, socioeconomic burden and health-related quality of life in rare disease patients in Europe (BURQOL-RD). BURQOL-RD had evaluated the socioeconomic burden for 10 rare diseases in the EU, including Bulgaria (18, 19). Our study did, however, include only 8 of those 10 conditions due to small sample size for the other 2. Additionally, we defined disease burden as the total sum of direct non-health-care costs, productivity loss, and early retirement costs. First quartile, median, and third quartile of the mean annual socioeconomic costs per patient per rare disease defined the performance cases of this criterion, and their scores were proportionally assigned. Cases below first quartile (low disease burden) got no points at all, while cases above third quartile (high disease burden) received all points of the criterion.

### Weights and Performance Scores of Decision Criteria That Account for the Public Health Considerations

**Figure 4:**
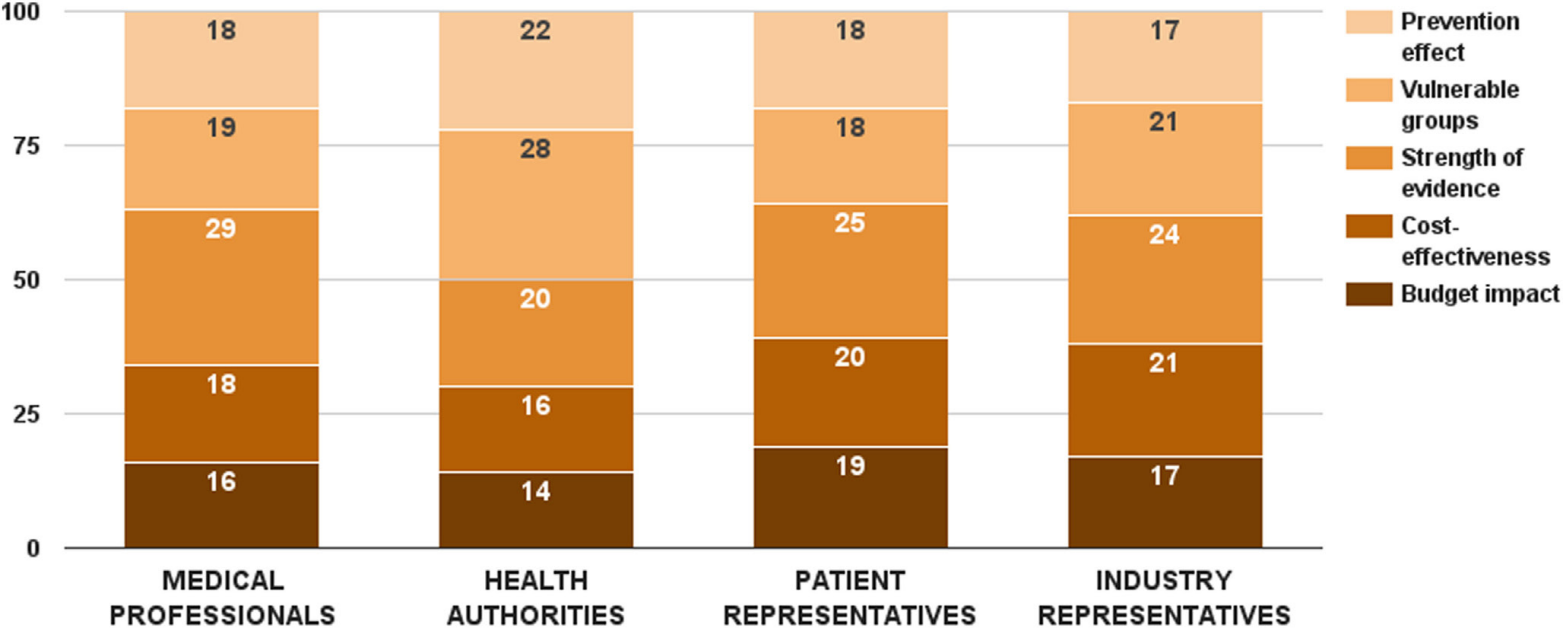
**Relative weight of decision criteria that account for the public health considerations per stakeholder groups**.

Survey participants considered strength of evidence as the most important decision criterion from this category. This criterion received 25 ± 10 value points out of 100. On the opposite side, budget impact was assessed as the least important decision criterion – 16 ± 9 points. While there was no significant difference in the weight values of budget impact and cost-effectiveness criteria per stakeholder groups, health authorities gave lowest points for both health economic criteria (Figure 4). There were two substantial differences in the distributed weight values from this decision criterion category. Medical professionals rated the evidence criterion significantly higher (*Z*_Kruskal–Wallis_ = 9.82, *p* = 0.020). So did health authorities on the vulnerable groups criterion (*Z*_Kruskal–Wallis_ = 9.56, *p* = 0.023). Individual values of all 5 criteria were normalized to sum up to the weight of the criterion category (24 points) – 4 points for budget impact, 5 points for cost-effectiveness, 6 points for strength of evidence, 5 points for vulnerable groups, and 4 points for prevention effect.

The budget impact criterion was constructed similarly to the disease burden criterion. Data for drug therapy costs were taken from the National Health Insurance Fund (20). First quartile, median, and third quartile of the mean annual drug therapy cost per patient per rare disease defined the cases of this criterion, and their performance scores were proportionally assigned. Cases below first quartile (low budget impact) got all the criterion weight, while cases above third quartile (high budget impact) received no points at all.

Cases for the cost-effectiveness criterion were differentiated through ICER. The scenario with more effects and less costs was the optimal performance score, receiving all 6 points. Choosing between the remaining two cases, stakeholders expressed preference for the scenario with more effects and more costs over the one with fewer effects and less costs – 4 vs. 1.

Different cases for the strength of evidence criterion were defined based on conventional hierarchy of evidence. Randomized controlled clinical trials constituted the highest performance score (8 points), while cross-sectional studies, case reports, and expert opinions got only 1 point each. Vulnerable groups criterion was defined with regard to the patient group that the health technology is indicated in. Prevention effect was a dichotomous variable, getting all the points in the maximum case and no points at all in the minimum case.

### Pilot Value Assessment and Appraisal Model

A total of 13 stakeholders (3 medical professionals, 4 health authority representatives, 3 patient representatives, and 3 industry representatives) participated in the focus group discussion. Three invitees declined to take part due to busy agenda. Nevertheless, they sent overall comments on the model. The meeting lasted 115 min.

The first half of the discussion focused on the list of criteria, weights, and performance scores. MCDA rationale was very positively perceived by all participants:

“MCDA gives an opportunity of associative thinking and in-depth analysis.” (Health authority representative)

“Innovative health technologies are not usually included in basic regulations and health insurance packages. Therefore, this issue requires many ad hoc decisions.” (Industry representative)

When reviewing weights and performance scores of decision criteria that describe the health technology’s characteristics, stakeholders debated the use of the alternative criterion in the context of rare diseases and lack of etiological treatment for those conditions. Patient representatives were adamant that in the absence of therapeutically equivalent alternatives, appraisal of orphan drugs has to be more implicit, and access of patients should be guaranteed. Other stakeholders accepted this rationale. Nevertheless, they pointed out that resources were scarce, and trade-offs were necessary:

“Health budget is always limited, resources can only be re-distributed. Re-directing resources to particular patients inevitably deprives others from necessary medical services.” (Health authority representative)

**Figure 5:**
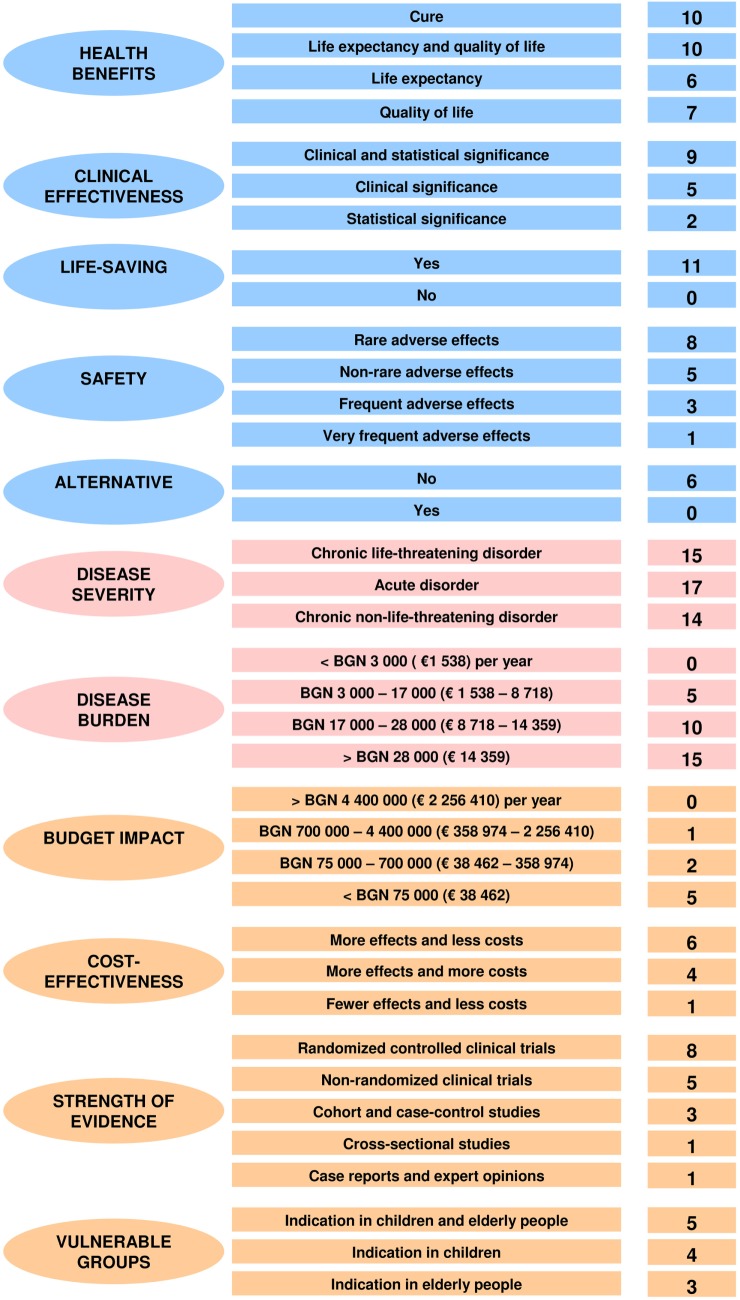
**Final weighted performance scores of the MCDA assessment and appraisal model for orphan drugs**.

There was a similar debate on the definition of the life-saving criterion. Stakeholders agreed that this condition is only met by emergency medicine health technologies. Overall, weights and performance scores of decision criteria that describe the health technology’s characteristics were consensually agreed, and no changes were made (Figure 5).

No amendments were needed for the weights and scores of decision criteria that describe the health-indicated disorder’s characteristics. All participants underlined that HTA and appraisal had to be put in the context of the disorders that these technologies were aimed at. There was no surprise that disease severity and disease burden were considered to be the most important decision criteria (Figure 5).

Weights and performance scores of decision criteria that account for the public health considerations were the most debatable part of the focus discussion. Strength of evidence was indeed accepted as a very substantial decision criterion. Nevertheless, physicians and health authorities took a position that real-world data from local settings had to be generated and collected too. Industry representatives defended data extrapolation as relevant, especially in case of rare diseases where expertise and experience were limited. Cost-effectiveness and budget impact were also a point of opposing views. Medical professionals, patients, and industry shared the opinion that access restrictions on innovative therapies had a strong negative impact on rare disease patients’ health. Patients went even further calling such decisions discriminatory. Physicians suggested that lack of resources was no excuse, and health-care system needed improving allocation of funds and spending control:

“I understand the rationale of health authorities that they are responsible for the efficiency of the health system. But there must be a way that allows patients to be treated and new evidence to be generated at the same time. Otherwise, it’s a vicious circle.” (Patient representative)

“I do not object to the economic justification of these restrictions. However, basic medical facts have to be taken into account. Such limitations must be consistent with the effect of treatment on the patient and should not be a financial goal solely.” (Medical professional)

The four representatives of health authorities involved in the discussion accepted those medical and ethical considerations but reminded that resources were limited and health-care system had to respond to growing health needs. The only change in the weights and performance scores of decision criteria that account for the public health considerations concerned the prevention effect criterion. Participants agreed that such a consideration was not applicable in this context. Furthermore, a health authority representative said that prevention effect was partially reflected by the criterion of health benefits. So, all stakeholders agreed to remove this decision criterion from the final MCDA value model and to transfer its weighting points to the other criteria from the same category (Figure 5).

**Figure 6:**
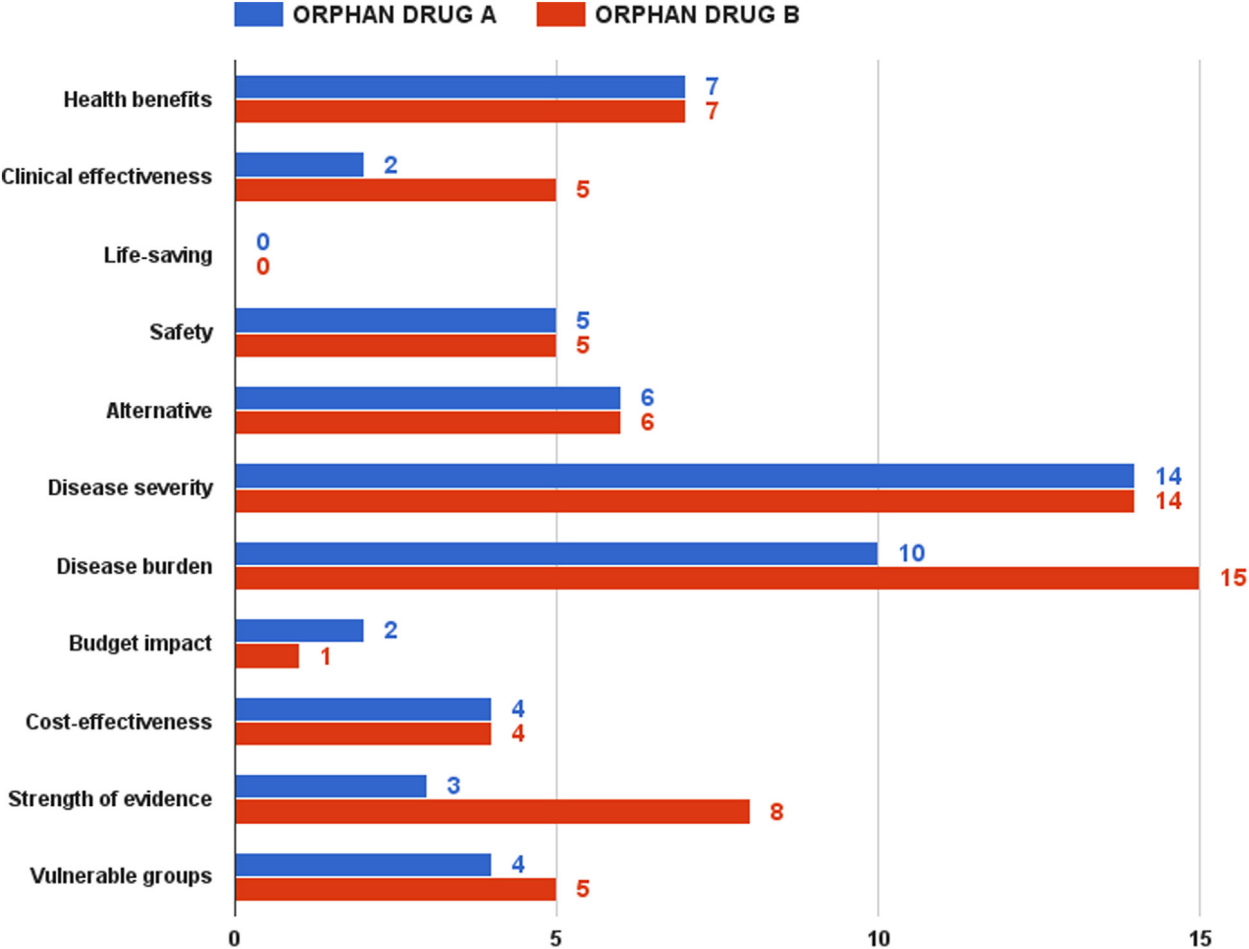
**Realistic MCDA assessment scenarios for orphan drugs A and B**.

The second half of the focus group discussion aimed to test the pilot model, as well as to decide upon a reimbursement recommendation threshold. Being acknowledged with the value assessment and appraisal model, as well as with the two hypothetical drugs, participants had to rate them. Overall, orphan drug B got higher scores than orphan drug A. This difference was, however, relatively small under the optimistic and pessimistic scenarios – 91 vs. 85 points and 52 vs. 44 points, respectively. This gap grew significantly in case of the realistic scenario, the most likely expected outcome of the assessment and appraisal process – 70 vs. 57 points (Figure 6).

Rarity, namely ultra rarity in case of orphan drug A, was a strong predictor of the final value estimate. If health benefits, life-saving, safety, and alternative criteria had similar performance scores in both drugs, the clinical effectiveness criterion achieved only 2 points in the realistic scenario for drug A against 5 points for drug B. Achieving clinical and statistical significance is considerably more difficult in ultra rare diseases, where the small number of patients makes it very hard to collect and analyze aggregated data. Although it was discussable to compare which of the two types of diseases – rare or ultra rare – would have a greater socioeconomic burden, participants shared the view that rare diseases, affecting both pediatric and adult cohorts, would have a bigger burden, given the loss or reduced productivity of both patient and main caregiver. In case of ultra rare pediatric disorder with low survival, these indirect costs would be lower. Public health considerations contributed for the final difference in values too – 18 vs. 13 points for drug B. The advantage for medicine B came almost entirely from the criterion of evidence strength – 8 vs. 3 points, taking into account the difficulty of conducting large-scale clinical trials in ultra rare diseases. As expected, budget impact was one of the few criteria, where orphan drug A dominated orphan drug B – smaller budget impact given the very small number of patients anticipated.

There was a wide range of proposals when discussing the threshold for a positive reimbursement decision. There was even a suggestion for a “floating” threshold depending on the allocated annual resources. Finally, stakeholders agreed on 70 or more points as a threshold for a positive recommendation (Table 3). In this context, a health technology that is rated between 50 and 70 points should be recommended for conditional reimbursement. As for health technologies receiving less than 50 points, participants backed the idea that this should not mean automatic rejection. Instead, industry could make concessions to ensure individual access schemes for rare disease patients.

**Table 3:** **Appraisal of the MCDA results**.

Result	Recommendation	Support tools
≥70 points (≥70%)	Unconditional reimbursement with public funds	Epidemiological registries
≥50 points (≥50%)	Conditional reimbursement with public funds	Epidemiological registries, risk-sharing agreements
<50 points (<50%)	No reimbursement with public funds	Individual access schemes

## Discussion

### Key Decision Criteria Preferences in Assessment and Appraisal of Orphan Drugs

Our study identified current perspectives on reimbursement decision-making in Bulgaria, as well as differences in stakeholders’ opinions. The methodological approach we applied allowed collection and analysis of what matters most in HTA. In a more specific context, this mixed-method research helped to understand variations on how orphan drugs are assessed and appraised with regard to their outcome for each decision criterion. Improved knowledge on MCDA feasibility and integration to HTA is of paramount importance, as progress in medicine and innovative health technologies should correspond to patient, health-care system, and societal values.

Decision criteria that describe the health technology’s characteristics were unanimously agreed as the most important group of reimbursement considerations. This outcome, combined with the high individual weight of disease severity and disease burden, underlined some of the fundamental principles of health care – equity and fairness. In this context, disease severity and disease burden are especially important decision criteria when their level is high. Reimbursement decisions should help relieve suffering in those who are worst off. There is increasing awareness that valuations of quality-adjusted life-years (QALYs) may differ when the QALYs accrue to different patients. Although these criteria are substantial in drug reimbursement decision-making, health authorities generally struggle in explicitly specifying the actual role and weight of disease severity and disease burden (21–23). Addressing this issue, Stolk et al. gave a valuable insight by describing the proportional shortfall approach that it used to operationalize disease severity in the Netherlands. Proportional shortfall concentrates on the fraction of QALYs that people lose relative to their remaining life expectancy, and not on the absolute number of QALYs lost or gained. So, it is the ratio of QALYs lost over the QALYs remaining (24). This is one possible equity adjustment to the standard cost-effectiveness criteria.

The economic criteria – cost-effectiveness and budget impact – turned out to be of lower importance for all stakeholders. Health authority representatives who are nominally responsible for the health-care system’s sustainability gave lowest weights to those criteria (although these variations were not statistically significant). Despite being a fundamental part of HTA, those considerations have been blamed for restricting patient access to treatment (11, 15). Cost-effectiveness is a leading indicator in priority setting and resource allocation. Meeting this criterion is the most important objective from a health economic perspective. In practice, however, not only orphan drugs but also very few innovative health technologies tend to be cost-effective. While various thresholds for ICER have been discussed, the application of this criterion remains a politically and morally dividing issue. ICER offers indeed a range of advantages, including reduced burden on decision-makers and consistency of decisions. Nevertheless, strict application of cost-effectiveness limits the possibility for flexibility and inclusion of *ad hoc* considerations in HTA (25). Moreover, explicit focus on ICER has been pointed out for marginalizing other important criteria, as well as for limiting patient choice and health-care rationing (26).

The budget impact criterion in reimbursement decisions has been similarly criticized. While economic analysis allows decision-makers to assess the effectiveness of health technologies, budget impact analysis is measuring the financial impact of the adoption and use of a new medical technology within the health system. This criterion is reflecting the accessibility of a new medical technology. Cost-effectiveness provides the basis for a favorable reimbursement decision, and budget impact determines what resources would be needed to actually implement this decision (27). Austerity makes regulators and payers more cautious about the impact that a new technology would have on their limited budget. With regard to orphan drugs, decision-makers fear that the costs would be significant and may cause changes in resource allocation. And this is what happens in practice, as health technologies with a high budget impact are much more likely to be rejected for reimbursement or to be subject of access restrictions (28).

Our study proved that strength of evidence may be a key criterion in MCDA. This was the top public health consideration, firmly supported by all stakeholders. Evidence is not only used to support reimbursement decision-making but also to lend legitimacy to policies pursued (29). Interpretation of evidence in decision-making is, however, influenced by several factors, such as organizational support, credibility, relevance, and applicability in practice, political support, and legislative constraints. Scarce or incomplete evidence definitely increases uncertainty around decisions. This could actually put new technologies at disadvantage in MCDA and reimbursement decisions (3). Our study showed that stakeholders are aware of the impact of rarity on orphan drugs’ evidence. They gave equal weight to cross-sectional studies, case reports, and expert opinions. The last two could partially replace clinical evidence at the initial stages of access. Nevertheless, all but industry representatives stressed the need for real-world data during the focus group discussion. In fact, national health systems have been increasingly looking into new coverage approaches to balance the need to provide adequate access to orphan drugs with the requirements to tackle uncertainty and obtain best value for money. Patient registries and risk-sharing agreements have been pointed out in particular for their potential with regard to this problem (30).

### Feasibility of MCDA Criteria in Assessment and Appraisal of Orphan Drugs

The fact that there are already several published studies exploring MCDA implementation in assessment and appraisal of orphan drugs suggests that this could be the most suitable approach for pricing and reimbursement for these medicinal products and innovative therapies in general. Back in 2013, Sussex et al. did one of the very first MCDA pilot studies for valuing orphan medicines. This particular model included eight non-monetary criteria – four concerning the disease being treated and four the treatment itself, with overall weights almost equally split between those two categories. Authors recommended developing such models for use by payers and HTA bodies (10). The most recent study of MCDA on orphan drugs was published in 2016 by Kolasa et al. An MCDA model was constructed, and assessment outcomes were compared with real-world reimbursement decisions in Polish context. Different results to the standard HTA process were detected. Authors suggested that further scrutiny of an orphan drug may increase the odds of a negative decision. Nevertheless, inclusion of more criteria was strongly supported (11). Extensive methodology analyses and systematic reviews have additionally supported this idea (12, 16, 17).

Determining a threshold for a positive reimbursement recommendation was one of the main tasks for our pilot model. While some authors recommend using MCDA estimates as a guide to decision-making rather than as a formula (3, 7), we believe that formalization of assessment and appraisal is a legitimate process, leading to balance and agreement among different stakeholders’ competing interests. Any negative decision is more acceptable whenever it is a result from a transparent and consistent framework (9). The study of Kolasa et al. considered MCDA outcome positive if more than 50% of the maximum number of points was reached. Otherwise, a negative result was assumed (11). Our focus group discussion yielded a rather different scenario (Table 3). Stakeholders consensually agreed on 70 or more points (corresponding to 70% at least from our value model) as a threshold for a positive recommendation. This may be significantly higher than the case of Kolasa et al. However, the only reasonable way to accept a higher valuation of orphan drug benefits is if these are demonstrated clearly and unequivocally (15). This strict threshold ensures that only orphan therapies with proven value for money profile are subject to reimbursement with public funds. This consideration was specifically outlined by the health authority representatives in our focus group discussion. They stressed on the overall sustainability of health-care system and reimbursement policy.

Our study participants agreed on a conditional reimbursement for a health technology rated between 50 and 70 points (Table 3). This is very much in line with the ongoing trend in reimbursement frameworks to link payment to results. In the context of fiscal austerity, timely access to innovative therapies has to be balanced against priorities and resources of the health system (30, 31). Risk-sharing agreements are built upon several factors, including balance between benefits and harms, certainty of evidence, resource considerations, values, and preferences. They represent performance-based reimbursement schemes, in which the price, level, or nature of reimbursement are tied to future performance measures of clinical or intermediate endpoints ultimately related to patient quality or quantity of life (32). Generation of new evidence is a key part of risk-sharing agreements, as real-world data on the therapy’s performance subsequently assist in making informed decisions on access. Patients and health professionals did endorse such option as a legitimate way to address rare disease patients’ unmet health needs.

Availability of alternative therapy (the alternative criterion) was an important moment in the two piloted hypothetical case studies. By legal definition in the EU, a medicinal product is orphan designated if (a) it is indicated in a rare condition, or it is indicated in a life-threatening, seriously debilitating, or serious and chronic condition, and that without incentives, it is unlikely that the marketing of the medicinal product would generate sufficient return to justify the necessary investment and (b) that there exists no satisfactory method of diagnosis, prevention, or treatment of the condition in question that has been authorized or, if such method exists, that the medicinal product will be of significant benefit to those affected by that condition (33). Patient representatives regarded this last part of the EU designation criteria for orphan drugs as a legal proof that these therapies had no alternatives. One patient participant even argued that there should be an automatic positive reimbursement recommendation in case of absence of therapeutic alternative. The choice or the lack of clinical comparator has been a contentious point in assessment and appraisal of orphan drugs (15). The decision here should be evidence-based, taking into account current clinical guidelines and unmet health needs.

### Limitations

Our study results should be considered in light of their limitations. We applied a simple linear additive approach to construct our MCDA value measurement model. It is acknowledged that there are more sophisticated methodologies, especially regarding weight and score elicitation (5, 12). Nevertheless, they pose a high degree of complexity and a significant cognitive burden for the participants. In discrete choice experiment studies that are very often recommenced in MCDA, the number of criteria and their levels are an important issue (7). This is why we opted for a simple, straightforward framework that allows for a comprehensive collection and analysis of preferences of various stakeholders on multiple criteria. Our approach is believed to be consistent with the way people usually made decision aggregations (11).

Selection of criteria is an important step in building an MCDA model. General MCDA methodology calls for completeness, non-redundancy, and mutual independence of decision criteria used (5). Our model does not fulfill the last of those requirements. Preferential independence means that decisions can be made by using a subset of criteria if the other criteria are the same for all alternatives irrespective of their actual values. That is, decisions can be made by using only the criteria on which the alternatives differ (5). From a practical point of view, this particular requirement is not feasible in health policy and health-care settings. These decisions are complex and need to balance various, sometimes conflicting, points of view. This is why we took and followed the preferences of Bulgarian stakeholders on the set of criteria for reimbursement decision-making. The incorporation of those opinions had definitely improved the external validity of our MCDA model.

Finally, our study was designed to address the Bulgarian health-care context. Preferences for weights and scores are country specific, as local resources, needs, and expectations strongly differ from one national health system to another. These differences impact the level of importance of individual criteria, making any value measurement model unique for its own public health settings (4). Nevertheless, our relative big sample could provide important insights for health policy stakeholders from various countries, as today all health-care systems face a common challenge – to balance limited budgets and increased expectations, formal requirements, and informal constraints (34, 35).

## Conclusion

Progress in medicine puts methodological pressure on HTA application in reimbursement decision-making. Standard HTA paradigm poses limitations regarding integration of the diversity of stakeholders’ perspectives. Conventional health economic tools do not allow responding to those concerns. MCDA could successfully address this challenge, as it provides a structured, transparent approach to identify preferred alternatives by means of combined calculation of the relative importance of different criteria and the performance of the alternatives on these criteria. MCDA framework enables the exploration of stakeholders’ preferences, as well as the explicit organization of broad range of criteria on which real-world reimbursement decisions are based.

Multi-criteria decision analysis potential is significant in the case of innovative health technologies and orphan drugs in particular. Assessment and appraisal of these medicinal products tend to be one of the most complicated and conflicting HTA tasks. This is why improved knowledge on MCDA feasibility and integration with HTA is of paramount importance, as the drive for innovation in medicine should match patient needs, health-care system resources, and societal values. Our study did create an MCDA value measurement model to assess and appraise orphan drugs in Bulgarian context, by exploring preferences on decision criteria’s weights and performance scores through a stakeholder-representative survey and piloting the model through a focus group discussion.

Decision criteria that describe the health technology’s characteristics were found to be the most important group of considerations in reimbursement decisions, making almost half of the total value estimate. This outcome, combined with the high individual weights of disease severity and disease burden criteria, underlined assessment, and appraisal of innovative therapies for rare diseases should be put in the context of the targeted condition. This conclusion was indirectly confirmed by the low weights of economic criteria. Our study proved that strength of evidence may be a key criterion in MCDA for orphan drugs. Scarce or incomplete evidence definitely increases uncertainty around decisions. So, patient registries and risk-sharing agreements are among the possible options to address this problem.

Despite the fact that preferences for weights and performance scores are country specific, our study’s relative big sample could provide important insights for health policy stakeholders from various countries. Today, all health-care systems face a common challenge – to balance limited budgets and increased expectations, formal requirements, and informal constraints.

## Author Contributions

All authors contributed to the preparation and publication of this paper.

## Conflict of Interest Statement

The authors declare that the research was conducted in the absence of any commercial or financial relationships that could be construed as a potential conflict of interest.
